# In vitro comparison of enamel shear bond strength to four restorative substrates used in resin-bonded prostheses

**DOI:** 10.1097/MD.0000000000050565

**Published:** 2026-09-11

**Authors:** Xing Wang, Jiaping Ma, Lu Qi

**Affiliations:** a First Affiliated Hospital of Xinjiang Medical University (Affiliated Stomatology Hospital), Urumqi, Xinjiang Uygur Autonomous Region, China; b School of Stomatology, Xinjiang Medical University, Urumqi, Xinjiang Uygur Autonomous Region, China; c The Second Affiliated Hospital of Xinjiang Medical University, Urumqi, Xinjiang Uygur Autonomous Region, China.

**Keywords:** enamel, failure-mode evaluation, resin-bonded bridge, restorative material, shear bond strength

## Abstract

The success of resin-bonded restorations relies on durable adhesion to enamel; however, the choice of restorative substrate may affect initial bonding behavior. This in vitro investigation evaluated and compared the micro-shear bond strength (SBS) to human enamel of 4 materials commonly used as substrates for resin-bonded prostheses: polyetherketoneketone (PEKK), cobalt–chromium (CoCr) alloy, zirconia ceramic, and a fiber-reinforced resin composite. Twenty-four extracted third molars with intact enamel surfaces were assigned to 4 groups (n = 6 per material). Standardized specimens measuring 5 × 5 × 3 mm were subjected to mechanical surface treatment by airborne-particle abrasion using 90-μm aluminum oxide at 0.30 MPa for 20 seconds, followed by ultrasonic cleaning. The specimens were then bonded to flattened enamel surfaces with an adhesive resin cement using a template that standardized the bonding area to a diameter of 1.5 mm. Following 24 hours of storage at 37°C, without thermocycling or fatigue loading, SBS was determined under monotonic loading at a crosshead speed of 0.5 mm/minute. Statistically significant differences in SBS were observed among the tested materials (*P* < .05). CoCr demonstrated the highest mean SBS (0.61 ± 0.20 MPa), followed by zirconia (0.36 ± 0.08 MPa), fiber-reinforced resin (0.29 ± 0.20 MPa), and PEKK (0.19 ± 0.08 MPa). CoCr showed significantly greater bond strength than all other groups, while zirconia also outperformed PEKK; no other pairwise comparisons reached statistical significance. Scanning electron microscopy revealed that failure patterns were predominantly mixed. Under these short-term, nonaged laboratory conditions, initial enamel bond strength differed by substrate type; these findings are limited to comparative laboratory adhesion behavior and should not be interpreted as evidence of long-term clinical performance or as a ranking of clinically optimized bonding protocols.

## 1. Introduction

Adhesive bonding of restorative materials to enamel is a major factor determining the outcomes of resin-bonded dental restorations. In vitro shear bond strength (SBS) tests are commonly performed by researchers in a controlled laboratory setting and can provide a means of evaluating the adhesive properties of a restorative material and comparing those properties directly, assuming all tests used a standardized testing protocol.^[1,2]^

Researchers have evaluated several different restorative substrates in adhesive dentistry, including cobalt–chromium (CoCr) alloys, zirconia ceramics, polyetherketoneketone (PEKK), and fiber-reinforced resin composites. Studies performing SBS tests have shown that the SBS of these materials to enamel varies from one another when bonded with resin cements, even when using similar adhesive systems.^[3–5]^ Additionally, the SBS values reported for these materials are highly variable across studies, a situation that is attributed to differences in specimen geometry, dimensions of bonded areas, methods of specimen surface preparation, and the types of test configurations used.^[6,7]^

Specifically, during laboratory studies, materials like PEKK and zirconia have demonstrated variable and, in some cases, lower SBS values to enamel, while CoCr alloy materials have consistently shown to produce higher SBS values for similar bonding and testing conditions. Materials made from fiber-reinforced resin composites have also been evaluated as methods of restorative substrates, yielding SBS results that frequently coincide with those of ceramic and polymeric materials.^[8]^ Although numerous SBS studies investigating the bonding properties of restorative substrates have been published, it is still difficult to compare indirectly across restorative materials due to differences in methodological techniques among studies.

The current literature does not provide standardized micro-SBS evaluation of adhesive bonding properties of multiple restorative materials under identical experimental protocols. Variations in the bond surface area, direction of the applied load, and specimen preparation have limited the ability to isolate the effect of material type on the bond strength of enamel.^[9]^

Therefore, this in vitro study has been designed to evaluate and compare the SBS of the following materials bonded to enamel using a standardized micro-shear test method, including CoCr, zirconia, fiber-reinforced resin, and PEKK. The null hypothesis states that there will be no difference in the SBS to enamel between these 4 restorative materials when they are tested under identical bonding and test conditions.^[10]^

## 2. Materials and methods

### 2.1. Materials

This study was approved by the Ethics Committee of The Second Affiliated Hospital of Xinjiang Medical University. The CoCr alloy, zirconia all-ceramic material, and PEKK specimens were sourced from Jiahong Dental Medical Co., Ltd. (manufacturer batch numbers were recorded at procurement). Fiber-reinforced resin specimens were obtained from Vactrise (batch number recorded). All bonding procedures were performed using RelyX Ultimate Adhesive Resin Cement (3M; lot number recorded). No additional primers, silane coupling agents, or chemical surface conditioners were used at any stage of the bonding process. Silicon carbide abrasive papers (220–2000 grit), super-hard dental gypsum, and polyethylene terephthalate sheets were purchased from Huiying Plastic Materials Co., Ltd. Surface roughening was performed using a JG-5833 airborne-particle abrasion unit (Wuhan Jinguang Medical Technology Co., Ltd.). Mechanical testing was conducted using a universal testing machine (Jinan Fangchen Instrument Equipment Co., Ltd.), and fracture surface evaluation was performed using a JSM-7610FPlus scanning electron microscope (JEOL).

### 2.2. Tooth selection

This study was approved by the Ethics Committee of the First Affiliated Hospital of Xinjiang Medical University, and written informed consent was obtained from all donors prior to tooth collection. Twenty-four freshly extracted human third molars were included. Only teeth with intact enamel surfaces, free from caries, restorations, cracks, hypoplastic defects, or visible structural damage were selected. Teeth showing enamel defects, surface contamination, or previous restorative procedures were excluded. No orthodontic history, craniofacial characteristics, radiographic parameters, facial symmetry criteria, or growth-related factors were considered. After extraction, the teeth were cleaned of residual soft tissue and stored in physiological saline at 4°C until specimen preparation.

### 2.3. Specimen preparation

The restorative materials tested were CoCr alloy, zirconia, PEKK, and a fiber-reinforced resin. Six specimens were produced for use with each type of material (a total of 24 specimens), and each specimen measured 5 mm × 5 mm × 3 mm. Bonding surfaces of all restorative specimens were prepared by airborne-particle abrasion with 90 μm aluminum oxide particles at a pressure of 0.30 MPa, a working distance of approximately 10 mm, and an application time of 20 seconds.

**Figure 1.**
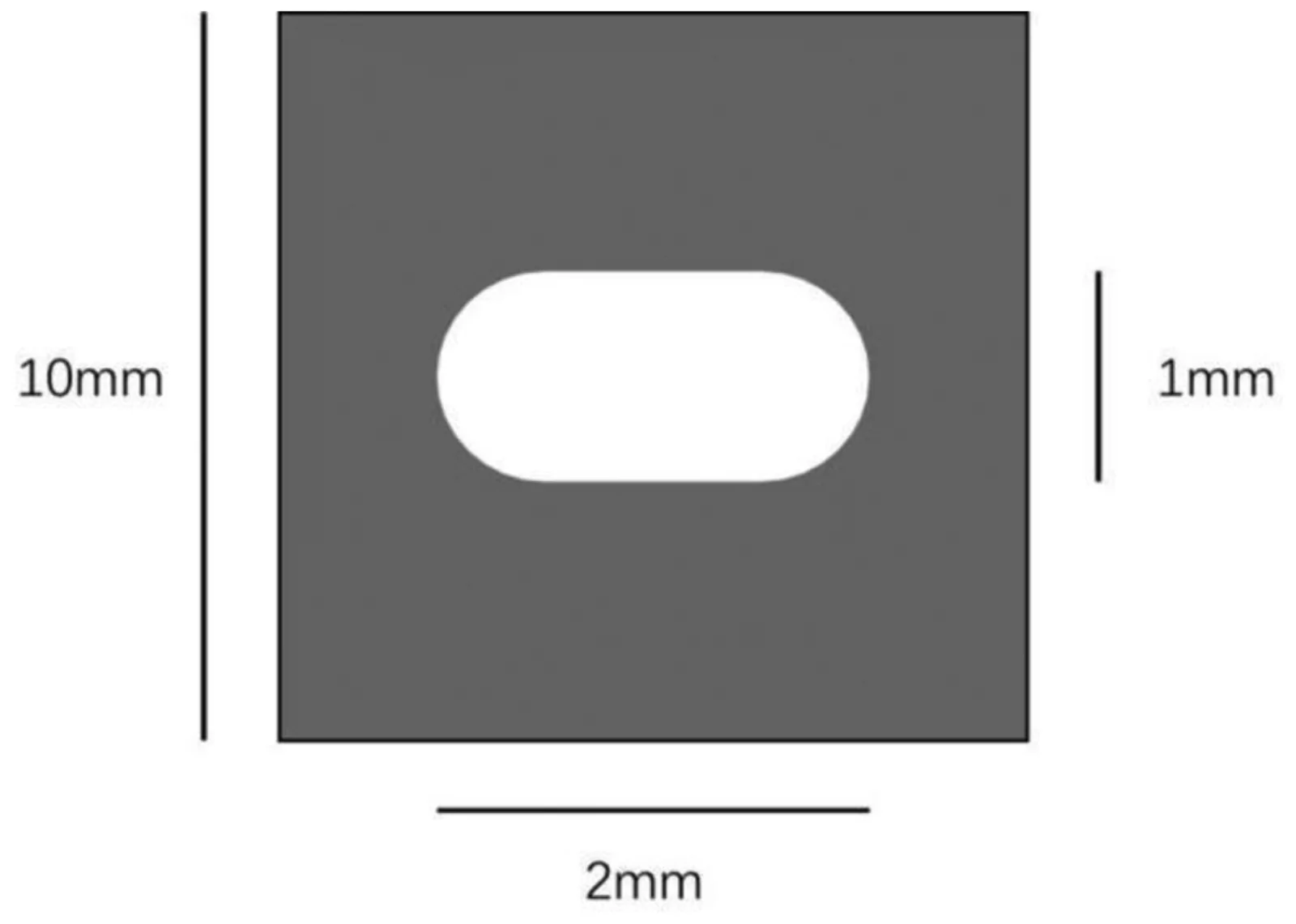
Representative surface morphology after sandblasting. Representative SEM images of cobalt–chromium, PEKK, zirconia, and fiber-reinforced resin specimens following airborne-particle abrasion with 90 μm aluminum oxide particles. Images were acquired at identical magnification; scale information is provided by the instrument settings. PEKK = polyetherketoneketone, SEM = scanning electron microscopy.

After sandblasting, all specimens were cleaned ultrasonically in ethanol and dried with compressed air. No chemical treatments, primers, or procedures for activating or chemically treating the surfaces were performed; thus, airborne-particle abrasion was the only restorative-surface pretreatment. This restriction was intentional because the primary experimental objective was to compare substrate-dependent bonding behavior while holding the surface-treatment protocol constant across all 4 materials. Material-specific primers, silane coupling agents, or chemical conditioners were therefore excluded to avoid introducing different chemical adhesion mechanisms as an additional experimental variable. This standardized design does not represent an optimized clinical bonding protocol for each substrate. Photographs showing representative surface morphologies after sandblasting are provided in Figure 1.

### 2.4. Bonding procedure

All teeth were polished under continuous water cooling using progressively finer silicon carbide abrasive papers (220–2000 grit) to create a flat enamel bonding surface. Each tooth was embedded in a 10 mm × 10 mm × 10 mm block of super-hard gypsum with the enamel surface exposed. A custom polyethylene terephthalate sheet with a thickness of 0.1 mm and a circular opening was positioned on the enamel surface to standardize the bonding area to 1.785 mm^2^ (corresponding to a diameter of 1.5 mm). Bonding was performed using RelyX Ultimate resin cement according to the manufacturer’s instructions. Light curing was carried out with an light emitting diode curing unit at approximately 1200 mW/cm^2^ for 20 seconds, with a curing distance of 1 to 2 mm from the enamel surface. This experimental configuration was designed exclusively to evaluate the enamel–material adhesive interface and does not simulate a Maryland bridge or any clinical fixed partial denture condition. After bonding, specimens were stored at 37°C for 24 hours prior to testing.

### 2.5. SBS testing

**Figure 2.**
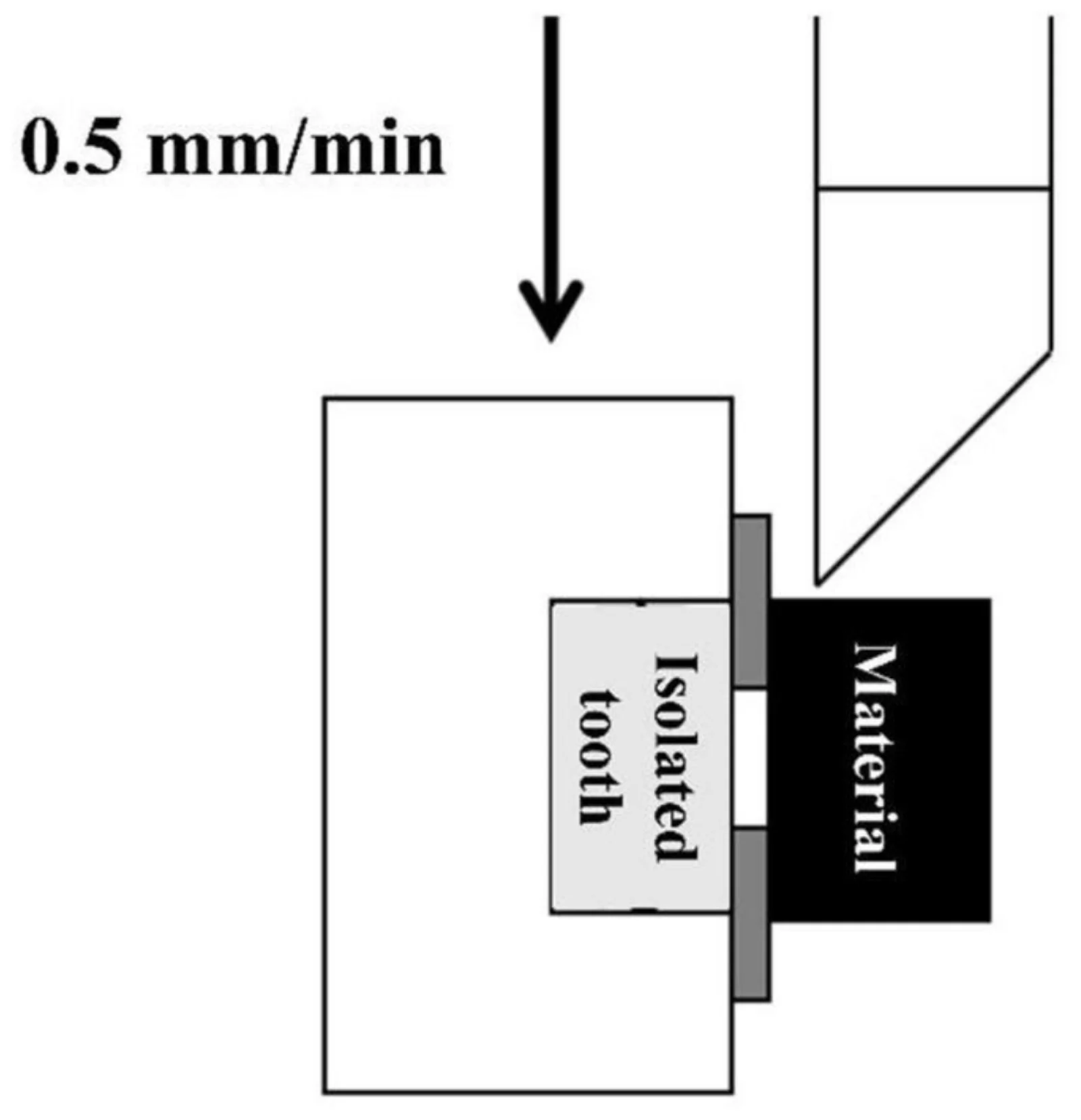
SBS testing setup. Experimental configuration showing specimen positioning within the universal testing machine during SBS testing. SBS = shear bond strength.

**Figure 3.**
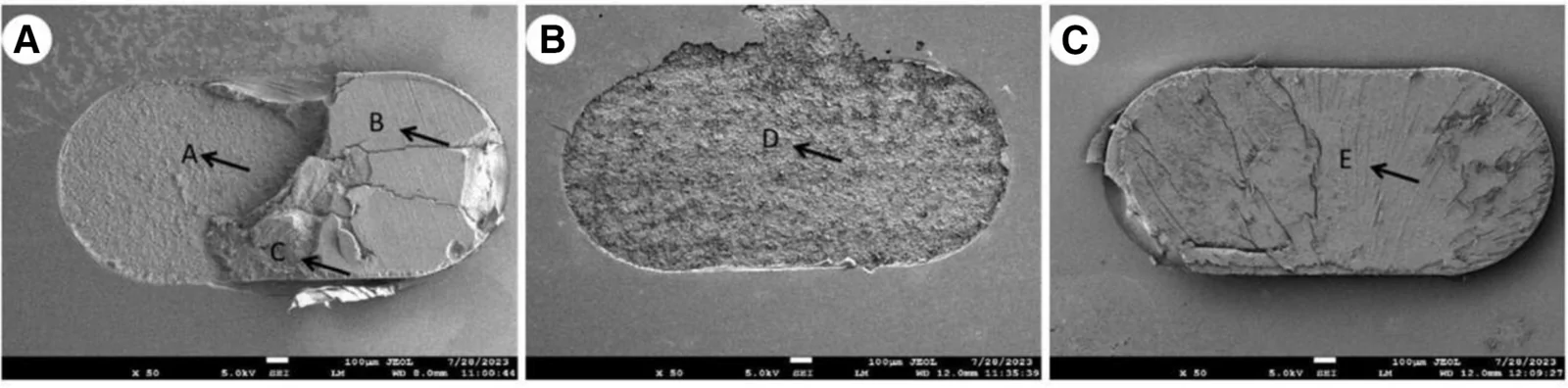
Shear force application during testing. Representative scanning electron micrographs (× 50) illustrating the gross fracture morphology observed after load-to-failure at the enamel–material interface. (A–C) show typical macroscopic failure appearances, including regions of exposed substrate and residual bonded components. The annotated arrows/labels indicate representative zones within the fractured area: (A) and (B) denote areas of exposed substrate with distinct surface texture; (C) highlights a localized region with residual adherent material consistent with mixed-mode failure; (D) indicates an extensively roughened fractured region; and (E) marks a crack-dominated region with visible fracture lines. Scale bar = 100 μm (as displayed).

SBS testing was performed using a universal testing machine equipped with a chisel-shaped loading head. The testing configuration and load application orientation are shown in Figures 2 and 3. Force was applied parallel to the enamel–material interface at a crosshead speed of 0.5 mm/min until debonding occurred. The maximum load at failure (N) was recorded and converted to SBS (MPa) by dividing the load at failure by the bonded surface area (1.785 mm^2^). All tests were conducted under static loading conditions only, with no thermocycling, mechanical fatigue, water aging, or environmental simulation applied. This testing protocol was used solely for comparative laboratory evaluation.

### 2.6. Fracture mode analysis

Following SBS testing, all specimens were initially examined under a stereomicroscope to assess general fracture characteristics, and fracture surfaces were subsequently examined using scanning electron microscopy (SEM) for morphological confirmation. Failure modes were classified according to the predominant location and appearance of the fractured area. Adhesive failure was defined as separation occurring primarily along the enamel–cement or cement–restorative-material interface with little or no cohesive fracture of either substrate. Cohesive failure in enamel was defined as fracture occurring predominantly within enamel while the bonded interface remained largely intact. Cohesive failure in the restorative material was defined analogously as fracture occurring predominantly within the restorative substrate. Mixed failure was assigned when both interfacial separation and cohesive fracture features were present in the same specimen. Fracture mode classification was descriptive only and was not used to infer bond quality, durability, or clinical performance.

### 2.7. Statistical analysis

Statistical analysis was performed using Statistical Package for the Social Sciences software, version 26.0 (IBM Corp.). SBS values were expressed as mean ± standard deviation. Normality and homogeneity of variance were assessed prior to analysis. Group comparisons were performed using 1-way analysis of variance (ANOVA), followed by Tukey post hoc test when appropriate. A significance level of *P* < .05 was applied for all statistical tests. The study included 6 specimens per group (total N = 24). A formal a priori power calculation was not performed before specimen testing. To clarify the statistical capability of this sample, a sensitivity analysis for a 4-group 1-way ANOVA at *α* = 0.05 indicated that N = 24 provides approximately 80% power to detect an omnibus effect size of *f* is approximately 0.74 or larger. Accordingly, the study was primarily capable of detecting large between-group effects; smaller differences could have remained undetected.

## 3. Results

### 3.1. SBS of different restorative materials

The SBS values of the 4 restorative materials are summarized in Table 1. The mean SBS values (mean ± standard deviation) were 0.61 ± 0.20 MPa for Group B (CoCr), 0.19 ± 0.08 MPa for Group A (PEKK), 0.36 ± 0.08 MPa for Group C (zirconia all-ceramic crown), and 0.29 ± 0.20 MPa for Group D (fiber-reinforced resin). One-way ANOVA demonstrated a statistically significant difference in SBS among the 4 groups (*P* < .05). Post hoc comparisons using Tukey test showed that Group B exhibited significantly higher SBS values than Groups A, C, and D (*P* < .05). Group C showed significantly higher SBS values than Group A (*P* < .05). No statistically significant differences were observed between Groups A and D or between Groups C and D (*P* > .05); given the limited group size, these nonsignificant comparisons should not be interpreted as evidence of equivalence.

**Table 1:** Comparison of shear strength of different repair materials.

Groups	Sample size	Shear strength (MPa)	
		Average	SD
PEKK	6	0.19	0.08
CoCr metal	6	0.61	0.20
Zirconia all-ceramic crown	6	0.36	0.08
Fiber-reinforced resin	6	0.29	0.20
Total	24	0.36	0.21

CoCr = Cobalt–Chromium, PEKK = polyetherketoneketone, SD = standard deviation.

### 3.2. Fracture modes observed by SEM

**Figure 4.**
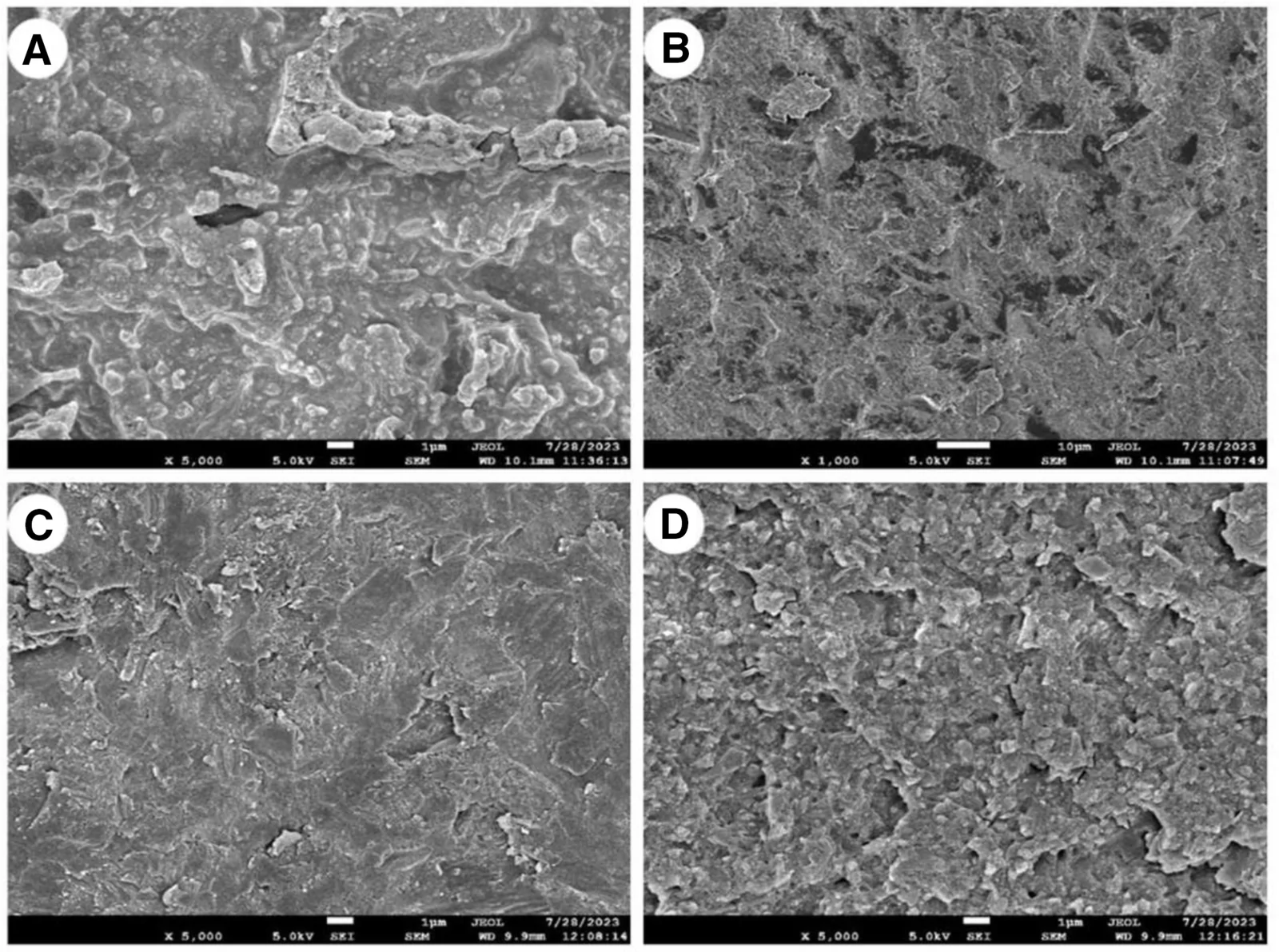
SEM image of the fracture surface of CoCr. Representative SEM micrographs of the debonded surfaces obtained after shear testing demonstrating microscale features associated with fracture and residual material distribution. Panels correspond to the 4 restorative material groups: (A) CoCr alloy; (B) fiber-reinforced resin; (C) zirconia ceramic; and (D) PEKK. The images illustrate heterogeneous surface microrelief with residual adherent phases and irregular fracture features consistent with predominantly mixed failure patterns identified by stereomicroscopic assessment. CoCr = Cobalt–Chromium, PEKK = polyetherketoneketone, SEM = scanning electron microscopy.

The distribution of fracture modes observed after SBS testing is presented in Table 2 and Figure 4. Among the 24 specimens tested, nineteen specimens exhibited mixed fracture patterns, 2 specimens showed cohesive failure within enamel, 2 specimens showed cohesive failure within the restorative material, and 1 specimen demonstrated adhesive failure at the bonding interface. No statistically significant differences in fracture mode distribution were detected among the 4 experimental groups (*P* > .05).

**Table 2:** Comparison of different fracture modes of various bonding specimens.

Groups	Fracture modes			
	Cohesive fracture of enamel	Cohesive fracture of materials	Bonded interface fracture	Mixed fracture
PEKK	0	1	1	4
CoCr metal	1	1	0	4
Zirconia all-ceramic crown	0	0	0	6
Fiber-reinforced resin	1	0	0	5

CoCr = Cobalt–Chromium, PEKK = polyetherketoneketone.

## 4. Discussion

This in vitro study compared enamel SBS among 4 restorative substrates using the same specimen geometry, bonding procedure, mechanical surface pretreatment, and static loading protocol. Statistically significant material-dependent differences were observed: CoCr showed the highest mean SBS, followed by zirconia, fiber-reinforced resin, and PEKK. These results describe relative initial adhesion behavior within the present nonaged laboratory model and should not be extrapolated directly to clinical longevity or to substrate performance under optimized material-specific bonding protocols.^[11–16]^

The relatively low SBS values observed across all 4 groups should be interpreted in the context of the experimental configuration rather than as direct evidence of poor clinical bonding performance. The small bonded area, monotonic static shear loading, and absence of thermocycling, water aging, or mechanical fatigue constrain the interpretation of the measured values.^[17,18]^ Thus, the present data are method-dependent short-term laboratory measurements.

The decision to exclude chemical surface treatments also affects the interpretation of the results. The same airborne-particle abrasion protocol was intentionally applied to all 4 substrates to isolate the effect of substrate type and to avoid confounding the comparison with material-specific chemical adhesion strategies. In clinical or material-optimization studies, however, primers, silane coupling agents, functional monomers, or other surface conditioners may enhance bonding to CoCr, zirconia, polymeric substrates, or fiber-reinforced composites to different extents.^[19,20]^ Their exclusion may therefore have reduced absolute SBS values and may have affected the relative performance of individual materials. Consequently, the present ranking should be understood only as the outcome of a common mechanical pretreatment protocol, not as a comparison of the best available clinical bonding protocol for each material.

The analysis of fracture patterns was included as a descriptive secondary outcome. Most specimens showed mixed failure, reflecting the coexistence of interfacial separation and cohesive fracture features under the present static shear configuration. Because failure-mode distributions can be influenced by stress concentration, specimen geometry, and test configuration, they should not be interpreted as direct indicators of bond durability or clinical performance.^[21]^ The revised classification criteria are therefore based only on the predominant fracture location and morphology observed by stereomicroscopy with SEM confirmation.

The focus of this research was to examine SBS in relation to static shear testing under controlled laboratory conditions. Factors affecting the long-term stability and/or degradation of bonded materials within the mouth, including cyclical loading, thermal cycling, and exposure to moisture, were not evaluated.^[22]^ The findings relating to this study are not representative of the clinical long-term performance of the tested bonded enamel materials, nor should the results be used to estimate the duration of adhesive-bonded materials on enamel surfaces. Future studies should incorporate aging procedures as part of the in vitro testing protocol; this would include thermal cycling, long-term water immersion, and/or cyclical loading of the adhesive-bonded interface,^[22,23]^ likely resulting in the development of data to enhance the static SBS data and improve the clinical value of the laboratory research studies.

## 5. Conclusion

Under the short-term, nonaged static laboratory conditions used in this in vitro study, SBS to enamel differed among the 4 restorative substrates, with CoCr exhibiting the highest mean value, followed by zirconia, fiber-reinforced resin, and PEKK. These findings are restricted to relative initial adhesion behavior under a standardized mechanical pretreatment protocol. Because no thermocycling, water aging, fatigue loading, clinical simulation, or material-specific chemical surface treatment was performed, the results should not be interpreted as predictive of long-term clinical performance or as a ranking of clinically optimized bonding strategies.

## Author contributions

**Conceptualization:** Xing Wang, Jiaping Ma, Lu Qi.

**Data curation:** Xing Wang, Jiaping Ma, Lu Qi.

**Formal analysis:** Xing Wang, Jiaping Ma, Lu Qi.

**Funding acquisition:** Lu Qi.

**Investigation:** Lu Qi.

**Writing – original draft:** Lu Qi.

**Writing – review & editing:** Lu Qi.
